# Construction and validation of a risk prediction model for perianal infection in HSCT patients: a retrospective cohort study

**DOI:** 10.3389/fonc.2026.1719205

**Published:** 2026-03-19

**Authors:** Xia Wu, Yan Gao, Chongying Yang, Haixia Ma, Pa Hu, Jianchuan Deng, Yin Liu, Xiuni Gan

**Affiliations:** 1 Department of Hematology, The Second Affiliated Hospital of Chongqing Medical University, Chongqing, China; 2 Evidence-based Nursing Research Center, The Second Affiliated Hospital of Chongqing Medical University, Chongqing, China; 3 Pediatric Department, The First Affiliated Hospital of Chongqing Medical University, Chongqing, China

**Keywords:** diarrhea, hematopoietic stem cell transplantation, neutropenia, perianal infection, prognostic nutritional index

## Abstract

**Background:**

Perianal infection is a frequent and detrimental complication in patients undergoing hematopoietic stem cell transplantation (HSCT), leading to significant morbidity and extended hospital stays. Despite its clinical significance, a substantial gap remains in our ability to proactively identify high-risk individuals. However a validated predictive model is actually lacking and it is still unclear how to identify these patients before the onset of infection.

**Objective:**

This study aimed to develop and to validate a risk prediction model for the accurate assessment of perianal infection risk following HSCT.

**Methods:**

A total of 353 patients who underwent HSCT between January 2020 and June 2023 were retrospectively enrolled in this study. The dataset was randomly split into a training cohort (n = 247) and a validation cohort (n = 106) at a ratio of 7:3. Univariate and multivariate logistic regression analyses were employed to identify independent risk factors. Based on these factors, a nomogram prediction model was constructed. Restricted cubic splines were used to assess nonlinear relationships. Model validation included discrimination (AUC), calibration (Hosmer-Lemeshow test), and clinical utility (decision curve analysis).

**Results:**

The independent risk factors of perianal infection following HSCT are the type of HSCT, history of perianal disease, diarrhea, neutropenia length, and preoperative prognostic nutritional index. In the training group, the model achieved an AUC of 0.846 (95% CI: 0.775-0.918), an accuracy of 81.0%, a sensitivity of 77.1%, a specificity of 81.9%, and a Youden’s index of 0.590. Subsequently, the validation group was assessed, showing an AUC of 0.829 (95% CI: 0.696-0.963), an accuracy of 73.6%, a sensitivity of 87.5%, a specificity of 71.1%, and a Youden’s index of 1.586. Model validation showed good calibration agreement. Decision curve analysis demonstrated clinically meaningful net benefit across a wide range of probability thresholds. Restricted cubic spline analysis revealed nonlinear associations of neutropenia length and preoperative PNI with perianal infection risk, with threshold analysis identifying clinically relevant inflection points.

**Conclusion:**

This study has constructed a simple, feasible, and visual nomogram for evaluating and early warning of perianal infection following HSCT.

## 1 Introduction

Hematopoietic stem cell transplantation (HSCT) is a standard treatment of for various hematologic disorders (1). However, HSCT preconditioning drugs and immunosuppressive therapy can lead to bone marrow suppression, decreasing immune function, and mucosal barrier damaging in patients, resulting in early postoperative infection being one of the main causes of death in HSCT patients (2).

For these reasons, perianal infection is a common complication following HSCT, especially in patients undergo allogeneic HSCT (allo-HSCT), which primarily occurs in the neutropenic period (3). According to the pathological stage, perianal infection may manifest as perianal abscess, anal fistula, and even necrotizing fasciitis, causing significant swelling, defecation disorders, severe pain, and even systemic infection, which may become life-threatening (4). Studies have reported that the cumulative incidence of perianal infection in allo-HSCT patients after transplantation is 15.3%-35.1% (5). Additionally, mortality in HSCT patients is significantly associated with perianal infection (6). Due to the anatomical location, perianal infection is easily overlooked in the early stages. Furthermore, disease progression often impact the quality of life of patients, leading to prolonged hospital stays and increased mortality rates.

Previous studies have demonstrated that perianal infection following HSCT is associated with a variety of factors, including sex (7), the type of HSCT (5), pre-transplant disease status (8), prior perianal history (5), history of constipation or diarrhea (9), chemotherapy exposure (10), neutropenia and hyperglycemia (11), but the systematic risk assessment tools or systematic models are lacking. Therefore, a validated prediction tool is required for early identification of HSCT patients at high risk of perianal infection, facilitating targeted preventive interventions. We aimed to construct a multivariate predictive model integrating known and newly identified risk factors to enable timely intervention and enhance prognostic outcomes. Furthermore, we aim to provide a systematic causal path framework to better understand the interactions among these risk factors and their causal relationship with perianal infections, which is intended to integrate clinical prediction indicators as measurable indicators of these core disorders, guiding our clinical model practice and creating a tool with biological interpretability.

## 2 Materials and methods

### 2.1 Study participants

This retrospective study included patients who underwent HSCT admitted to the Department of Hematology in the Second Affiliated Hospital of Chongqing Medical University from January 2020 to June 2023. This study collected data from 364 HSCT patients. Among them, 11 cases were excluded due to incomplete medical records, due to the extremely small missing ratio (<5%) and the situation of completely random missing (MCAR) (12). Thus, finally 353 patients were included for model development and validation. All patients received standardized perianal care (twice-daily disinfection with povidone-iodine) without routine systemic antibiotic prophylaxis. Utilizing the random number table technique, the registered patients were divided into two groups: a training group (n = 247) and a validation group (n = 106), based on a ratio of 7:3 (11). The training group was used for model construction, while the validation group was used for model validation. Our analysis was limited to the initial instance of perianal infection in patients throughout the duration of the study. This study received ethical approval from the Hospital Ethics Committee, and all researchers conducted the research in accordance with the ethical guidelines of the Helsinki Declaration (Approval No. 2024-198).

### 2.2 Inclusion and exclusion criteria

Inclusion criteria were: (1) Patients received HSCT with myeloablative preconditioning protocols; (2) Patients were 14–65 years old; patients had not perianal infection before pretreatment; (3) Perianal infection occurred within 100 days after HSCT. Exclusion criteria: (1) Treatment interruption or change of departments due to illness; (2) More than 100 days after HSCT; (3) Lack of important clinical data, (4) Incomplete medical history or examination data.

### 2.3 Diagnostic criteria

The diagnosis of perianal infection was determined by inpatient medical records, which were established by hematologists and specialists in anorectal diseases. Perianal infection was defined and diagnosed by new pain and at least two of the following manifestations: erythema, swelling, tenderness, sclerosis, and fluctuation. The evaluation criteria for perianal infection were as follows: Grade I, local inflammation such as redness, swelling, fever, and pain; Grade II, local inflammation with abscess formation; Grade III, skin ulceration, necrosis, bleeding, sinus or fistula formation (5).

### 2.4 Study variables and data collection

This study employed a comprehensive approach involving literature review, expert consultation, and discussion with the research team to identify potential risk factors for perianal infection in HSCT patients. Consequently, a total of 12 independent variables were included and analyzed in this study, including sex, age, BMI, hematologic disorders, the type of HSCT, neutropenia length, history of perianal disease, preoperative albumin concentration, preoperative prognostic nutritional index (PNI), diarrhea, diabetes, intestinal infection. The dependent variable in this study was perianal infection.

Based on the dependent variable and independent variables determined by the research team, a data collection form was designed for patients with perianal infection following HSCT, and the relevant data were collected from electronic medical records by two investigators. Researchers collected the relevant data from medical records, including sex, height, weight, age, hematologic disorders, the type of HSCT, neutropenia length, preoperative albumin concentration, preoperative lymphocyte counts, history of perianal disease, history of diabetes, diarrhea, intestinal infection and whether perianal infection. Thereafter, patients’ body mass index (BMI) was calculated based on the collected data (13). Of note, PNI is a commonly used index that reflects the nutritional status and immune function of patients, which is calculated according to the serum albumin concentration and the total number of peripheral lymphocytes (14). The PNI was calculated according to the formula PNI = serum albumin (g/L) + 5 × lymphocyte count (10^9^/L) (15).

### 2.5 Statistical analysis

SPSS (V.25.0) and R Statistical Software (V.4.3.3) were used for statistical analysis in this study. Due to the extremely small missing ratio (<5%) and completely random missing (MCAR) cases, the cases (rows) containing missing values were directly removed. Patients with missing data were excluded, and only complete cases were included in the final analysis. Quantitative data with a normal distribution are expressed as mean ± standard deviation ( $\bar{x}\pm s$), and the independent-samples t−test is used for comparisons between groups. Quantitative data with a non−normal distribution are presented as median (Q1, Q3), and the Wilcoxon rank−sum test (also known as the Mann−Whitney U test) is used for group comparisons. Categorical variables are reported as counts and percentages (%), and group comparisons are performed using the chi−square test. When the sample size is small (n < 40) or the expected frequency in any cell is insufficient, Fisher’s exact test is applied instead. Statistically significant variables in the univariate analysis were included in the binary logistic regression analysis, and variables with *p* > 0.05 in the multivariate analysis were determined as independent predictors of perianal infection. To evaluate the assumption of collinearity, the variance inflation factors (VIF) were computed, considering a VIF below 5 as a sign of insignificant collinearity. Furthermore, illustrate the restricted cubic splines (RCS) curve to investigate the non-linear correlation between the continuous variables and perianal infection. Threshold analysis was performed to identify optimal risk cut-offs for clinical intervention. The final nomogram was constructed using the ‘rms’ package in R software (version 4.3.3). Internal validation was performed via bootstrap resampling with 1,000 iterations to assess model performance. The effectiveness of the model was assessed using the receiver operating characteristic (ROC) curve (area under the curve AUC), calibration plot, Hosmer-Lemeshow goodness-of-fit tests, and decision curve analysis (DCA). *p<* 0.05 was considered statistically significant.

## 3 Results

### 3.1 General information and characteristics

This study collected data from 364 HSCT patients. Among them, 11 cases were excluded due to incomplete medical records, and finally 353 patients were included for model development and validation. The participants included 171 (48.4%) men and 182 (51.6%) women. Specifically, the cohort included 11 (3.1%) cases of myelodysplastic syndrome, 84 (23.8%) cases of multiple myeloma, 89 (25.2%) cases of lymphoma, 135 (38.3%) cases of leukemia, 21 (6.0%) cases of aplastic anemia, and 13 (3.6%) other cases. Among them, 117 (33.1%) patients received allo-HSCT, and 236 (66.9%) patients received autologous hematopoietic stem cell transplantation (auto-HSCT). Age distributions were similar between the training group (47.19 ± 14.96 years) and validation group (47.97 ± 15.12 years). The training group included 247 patients with HSCT, among whom 19.43% (48/247) developed perianal infections. Notably, the cohort included 38 cases of perianal soft tissue infection, 8 cases of perianal abscess, 1 case of anal fistula, and 1 case of anal fistula combined with necrotizing fasciitis. In the validation group, consisting of 106 HSCT patients, 16 patients developed perianal infections, yielding a prevalence rate of 15.09%, including 9 cases of perianal soft tissue infection and 7 cases of perianal abscess. Comprehensive baseline data and clinical characteristics were collected. This research compared distinct variables between the training and validation groups, with no statistically significant differences were observed, as illustrated in **Table 1** (*p* > 0.05).

**Table 1:** Baseline characteristics comparison between the training and validation group.

Variables	Total (n=353)	Training group (n=247)	Validation group (n=106)	P
Sex, n (%)				0.585
Female	182 (51.56)	125 (50.61)	57 (53.77)	
Male	171 (48.44)	122 (49.39)	49 (46.23)	
BMI, Median (Q1,Q3)	22.22 (20.03, 24.57)	22.19 (19.70, 24.56)	22.29 (20.25, 24.61)	0.477
Age, Median (Q1,Q3)	50.00 (38.00, 58.00)	50.00 (37.00, 58.00)	50.00 (41.00, 58.75)	0.716
Hematologic disorders, n (%)				0.747
Aplastic anemia	21 (5.95)	15 (6.07)	6 (5.66)	
Leukemia	135 (38.24)	97 (39.27)	38 (35.85)	
Lymphoma	89 (25.21)	58 (23.48)	31 (29.25)	
Multiple myeloma	84 (23.80)	59 (23.89)	25 (23.58)	
Myelodysplastic syndrome	11 (3.12)	7 (2.83)	4 (3.77)	
Else	13 (3.68)	11 (4.45)	2 (1.89)	
Type of HSCT, n (%)				0.599
Allo-HSCT	236 (66.86)	163 (65.99)	73 (68.87)	
Auto-HSCT	117 (33.14)	84 (34.01)	33 (31.13)	
Neutropenia length, Median (Q1,Q3)	15.00 (11.00, 21.00)	15.00 (12.00, 22.00)	14.00 (11.00, 20.00)	0.158
Preoperative albumin concentration, Median (Q1,Q3)	38.80 (35.50, 42.50)	38.90 (35.25, 42.50)	38.65 (36.10, 42.15)	0.723
Preoperative lymphocyte counts, Median (Q1,Q3)	0.76 (0.35, 1.51)	0.78 (0.36, 1.54)	0.72 (0.32, 1.33)	0.521
Preoperative PNI, Median (Q1,Q3)	44.05 (39.20, 50.70)	44.50 (39.38, 51.60)	43.58 (39.04, 48.75)	0.303
History of perianal disease, n (%)				0.301
No	310 (87.82)	214 (86.64)	96 (90.57)	
Yes	43 (12.18)	33 (13.36)	10 (9.43)	
History of diabetes, n (%)				0.619
No	311 (88.10)	219 (88.66)	92 (86.79)	
Yes	42 (11.90)	28 (11.34)	14 (13.21)	
Diarrhea, n (%)				0.090
No	280 (79.32)	190 (76.92)	90 (84.91)	
Yes	73 (20.68)	57 (23.08)	16 (15.09)	
Intestinal infection, n (%)				0.841
No	274 (77.62)	191 (77.33)	83 (78.30)	
Yes	79 (22.38)	56 (22.67)	23 (21.70)	

BMI, body mass index; HSCT, hematopoietic stem cell transplantation; PNI, prognostic nutritional index.

### 3.2 Analysis of perianal infection in the training group

As presented in **Table 2**, a total of 247 HSCT patients were included in the training group. The training group was subjected to univariate analysis, which incorporated 12 potential risk factors associated with perianal infections following HSCT. The analysis revealed no statistically significant differences in 6 items, including sex, BMI, Hematologic disorders, preoperative albumin concentration, intestinal infection, and history of diabetes (*p* > 0.05). Conversely, statistically significant differences were observed in age, the type of HSCT, neutropenia length, preoperative PNI, history of perianal disease, and diarrhea (*p<* 0.05).

**Table 2:** Single factor analysis of perianal infection in the training group.

Variables	Total (n=247)	Non-Perianal infection group (n=199)	Perianal infection group (n=48)	*P*
Sex, n (%)				0.820
Female	125 (50.607)	100 (50.251)	25 (52.083)	
Male	122 (49.393)	99 (49.749)	23 (47.917)	
BMI, Median (Q1,Q3)	22.189 (19.700, 24.563)	22.222 (19.642, 24.419)	22.167 (20.406, 25.352)	0.351
Age, Median (Q1,Q3)	50.000 (37.000, 58.000)	52.000 (40.000, 58.000)	44.500 (32.000, 53.000)	0.012
Hematologic disorders, n (%)				0.186
Aplastic anemia	15 (6.073)	11 (5.528)	4 (8.333)	
Leukemia	97 (39.271)	73 (36.683)	24 (50.000)	
Lymphoma	58 (23.482)	46 (23.116)	12 (25.000)	
Multiple myeloma	59 (23.887)	52 (26.131)	7 (14.583)	
Myelodysplastic Syndrome	7 (2.834)	6 (3.015)	1 (2.083)	
Else	11 (4.453)	11 (5.528)	0 (0.000)	
Type of HSCT, n (%)				< 0.001
Allo-HSCT	163 (65.992)	147 (73.869)	16 (33.333)	
Auto-HSCT	84 (34.008)	52 (26.131)	32 (66.667)	
Neutropenia length, Median (Q1,Q3)	15.000 (12.000, 22.000)	15.000 (11.000, 19.000)	21.500 (17.750, 26.250)	< 0.001
Preoperative albumin concentration, Median (Q1,Q3)	38.900 (35.250, 42.500)	38.900 (35.050, 42.300)	38.850 (36.125, 43.200)	0.490
Preoperative PNI, Median (Q1,Q3)	44.500 (39.375, 51.600)	45.200 (40.250, 53.350)	41.900 (26.862, 49.325)	0.004
History of perianal disease, n (%)				< 0.001
No	214 (86.640)	183 (91.960)	31 (64.583)	
Yes	33 (13.360)	16 (8.040)	17 (35.417)	
History of diabetes, n (%)				0.216
No	219 (88.664)	174 (87.437)	45 (93.750)	
Yes	28 (11.336)	25 (12.563)	3 (6.250)	
Diarrhea, n (%)				< 0.001
No	190 (76.923)	167 (83.920)	23 (47.917)	
Yes	57 (23.077)	32 (16.080)	25 (52.083)	
Intestinal infection, n (%)				0.668
No	191 (77.328)	155 (77.889)	36 (75.000)	
Yes	56 (22.672)	44 (22.111)	12 (25.000)	

BMI, body mass index; HSCT, hematopoietic stem cell transplantation; PNI, prognostic nutritional index.

### 3.3 Multivariate analysis of perianal infection in the training group

As illustrated in **Table 3**, a multivariate logistic regression analysis was conducted using perianal infection as the dependent variable and variables have shown statistical significance in the univariate analysis as independent variables. The analysis indicated that the type of HSCT, history of perianal disease, diarrhea, neutropenia length, and preoperative PNI were independent risk factors for perianal infection. Specifically, allo-HSCT was associated with an increased risk of perianal infection compared to auto-HSCT (OR: 3.347, 95% CI: (1.491-7.729, *p<* 0.05). Patients with history of perianal disease before transplantation exhibited a significantly higher risk of developing perianal infection following HSCT (OR: 5.608, 95% CI: 2.128-15.150, *p<* 0.05). Diarrhea was also associated with an increased risk of perianal infection (OR: 5.452, 95% CI: 2.402-12.737, *p<* 0.05). Neutropenia length was correlated with a greater risk of perianal infection (OR: 1.047, 95% CI: 1.015-1.083, *p<* 0.05). Additionally, lower preoperative PNI values were associated with a higher risk of perianal infection (OR: 0.943, 95% CI: 0.907-0.977, *p<* 0.05). The results of the collinearity test show that VIF values of all variables are less than 5, indicating no severe collinearity.

**Table 3:** Multivariate logistic regression analysis in the training group.

Variables	Coefficients	SE	Wald χ^2^	OR (95% CI)	*P*
Age	-0.014	0.014	-1.004	0.986 (0.96-1.014)	0.316
Type of HSCT	1.208	0.417	2.896	3.347 (1.491-7.729)	0.004
Neutropenia length	0.046	0.016	2.856	1.047 (1.015-1.083)	0.004
Preoperative PNI	-0.058	0.019	-3.101	0.943 (0.907-0.977)	0.002
History of perianal disease	1.724	0.497	3.471	5.608 (2.128-15.150)	0.001
Diarrhea	1.696	0.423	4.009	5.452 (2.402-12.737)	<0.001

SE, standard error; Wald χ^2^, Wald’s equation; OR (95 CI), Odds Ratio (95% Confidence Interval).

### 3.4 Construction of a clinical prediction model

**Figure 1:**
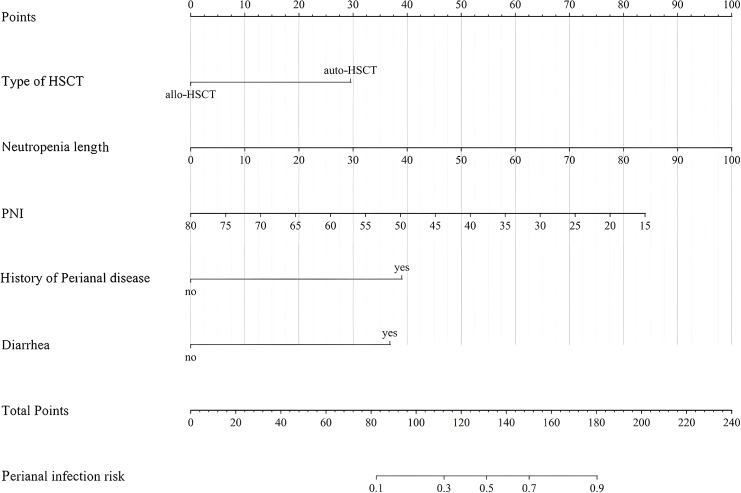
A nomogram model for risk of perianal infection in training group.

The clinical prediction model was established according to the results of the logistic regression analysis, including the type of HSCT, history of perianal disease, diarrhea, neutropenia length, and preoperative PNI as predictors and perianal infection as the clinical outcome (set as the binary dependent variable). Five distinct risk elements were input into the R software, utilizing the ‘lrm’ feature in the ‘rms’ package to construct the nomogram prediction model. Each factor’s value was ascertained by where the vertical line from the variable intersects with the point axis, and the aggregate risk score was computed by summing up all points of the variable. As shown in **Figure 1**, the patient’s risk of perianal infection can be assessed by adding the scores of all variables on the nomogram.

### 3.5 Validation of the prediction model

**Figure 2:**
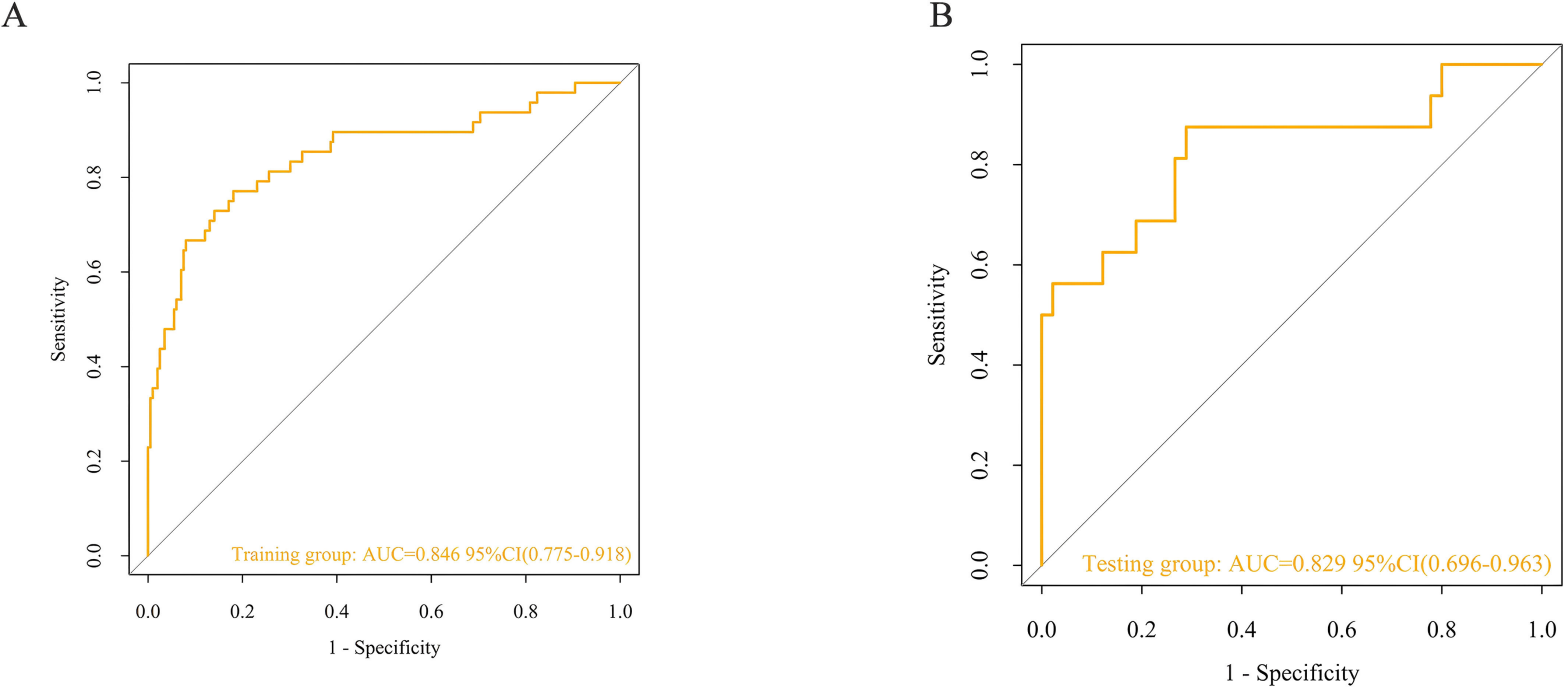
ROC curves in the training group **(A)** and the validation group **(B)**.

**Figure 3:**
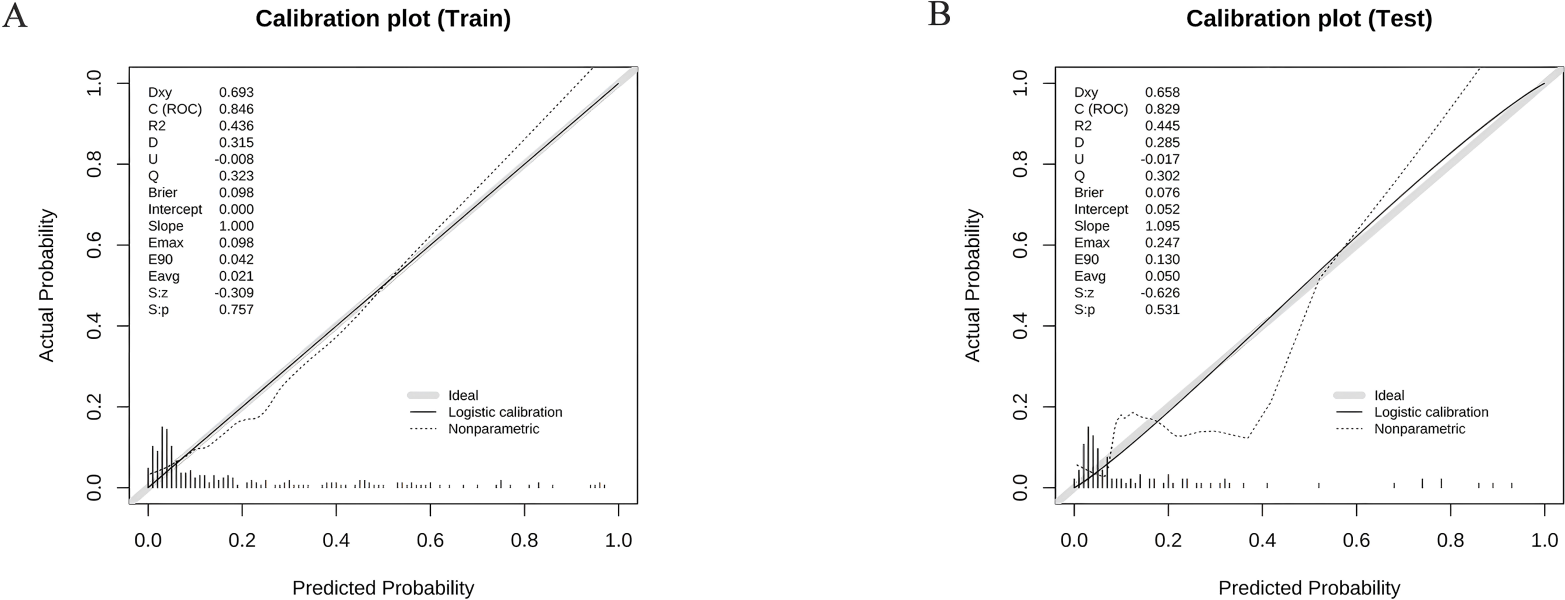
Calibration plots between the training group **(A)** and validation group **(B)**.

**Figure 4:**
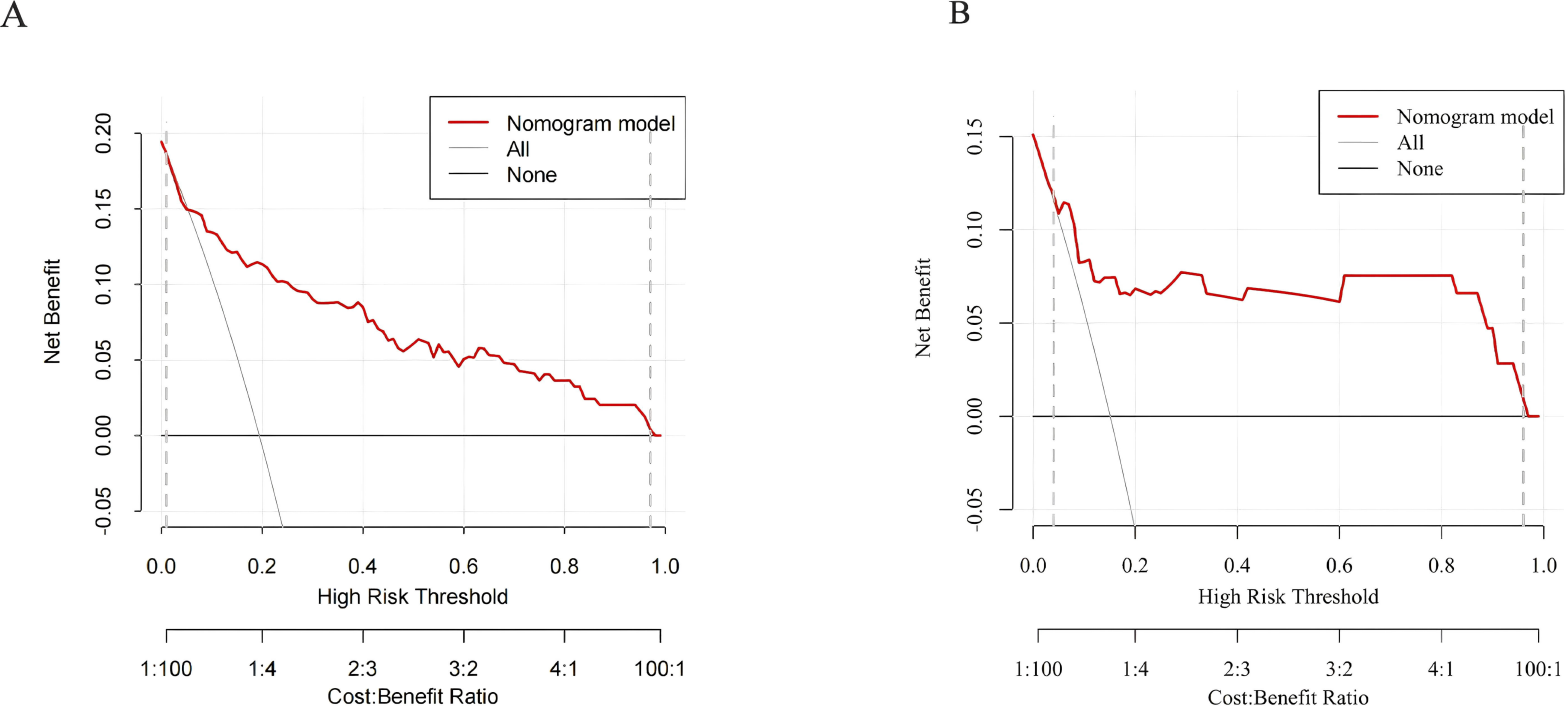
Decision curves for the training group **(A)** and validation group **(B)**.

In the training group, the results from 1000 internal bootstrap validations revealed an AUC of 0.846 (95% CI: 0.775-0.918), at a cut-off value of 0.204, a model accuracy of 81.0%, a sensitivity of 77.1%, a specificity of 81.9%, and a Youden index was 0.590. as displayed in **Figure 2A**. Moreover, the calibration plot showed that the predicted values of the model are in good agreement with the actual observed values, indicating that the model has a high calibration degree, as shown in **Figure 3A**. The Hosmer-Lemeshow test results indicated that the risk prediction model had a good fit (χ² = 11.322, *p* = 0.184). In the training group, the model demonstrated excellent calibration with a calibration slope of 1.000, a intercept of 0.000, and a Brier score of 0.098. To further evaluate the clinical utility of the model, a decision curve analysis was performed (**Figure 4**). As depicted in **Figures 4A**, the nomogram demonstrated the greatest overall advantage over threshold probabilities ranging from1-97% in the training group, supporting its application in clinical decision making. Furthermore, model achieved an AUC of 0.829 (95% CI: 0.696-0.963) in the validation group and exhibited a model accuracy of 73.6%, a sensitivity of 87.5%, a specificity of 71.1%, and a Youden ‘s index of 1.586, with the corresponding cut-off value of 0.103, as shown in **Figure 2B**. The calibration plot revealed a strong resemblance between the forecasted likelihood of the nomogram and the real probabilities of both the training and verification groups, as shown in **Figure 3B**. The Hosmer-Lemeshow goodness of fit test showed χ² = 17.53, *p* = 0.063, indicating a good model fit. The calibration metrics remained strong, with a slope of 1.095, an intercept of 0.052, and a Brier score of 0.076. This indicated that the calibration plot demonstrated a good consistency between the predicted value of the model and the actual value. The AUC was similar to that observed during the training group, indicating that the model has a high predictive accuracy for HSCT patients. In the validation group, the nomogram provided superior net benefit across a clinically relevant threshold probability range of 4-96%, as shown in **Figure 4B**. These findings indicate that it has good clinical efficacy in decision-making.

### 3.6 RCS and threshold analysis of neutropenia length and preoperative PNI

**Figure 5:**
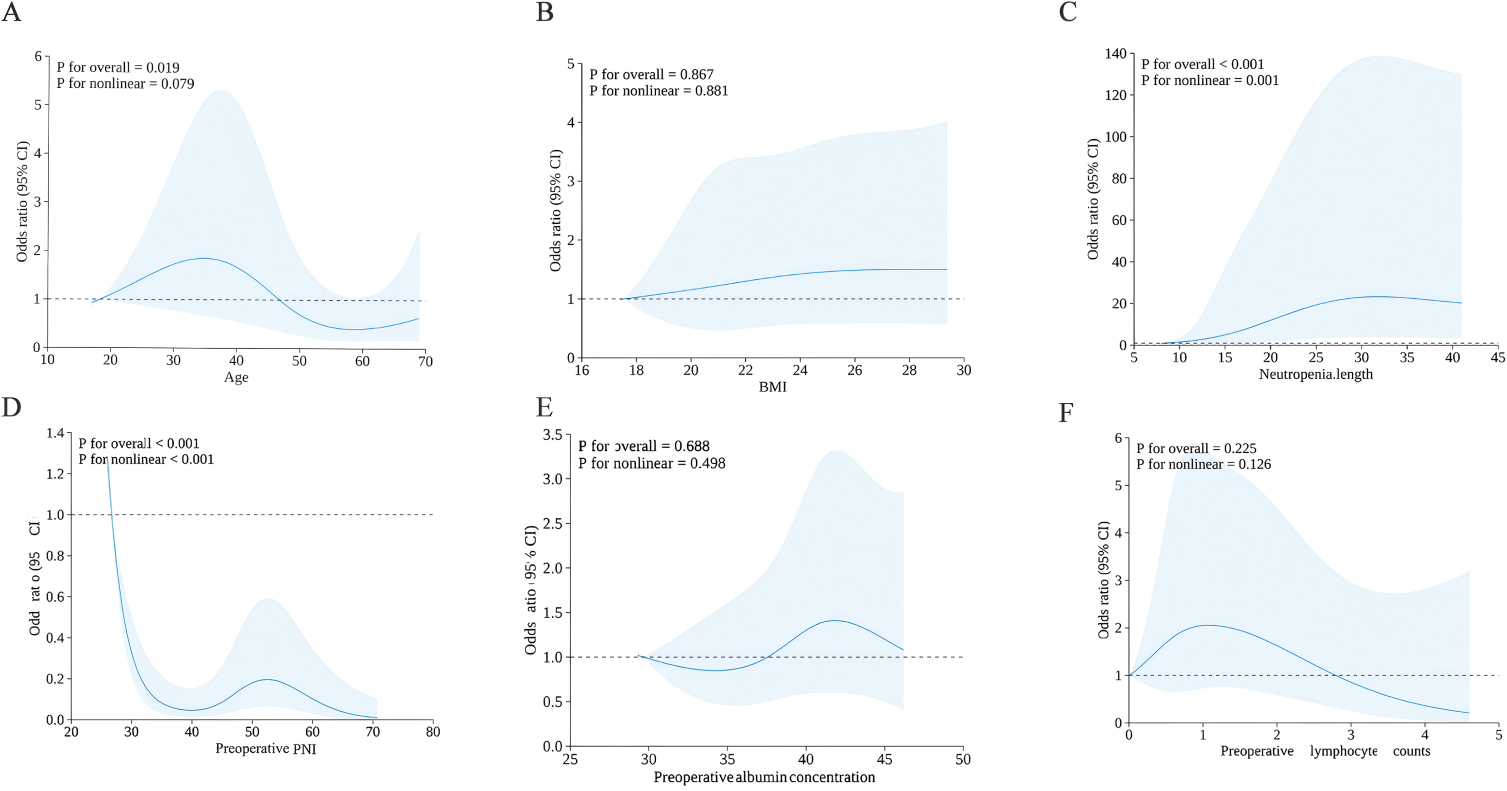
Restricted cubic spline analysis of neutropenia length, preoperative PNI and infection risk.

**Figure 6:**
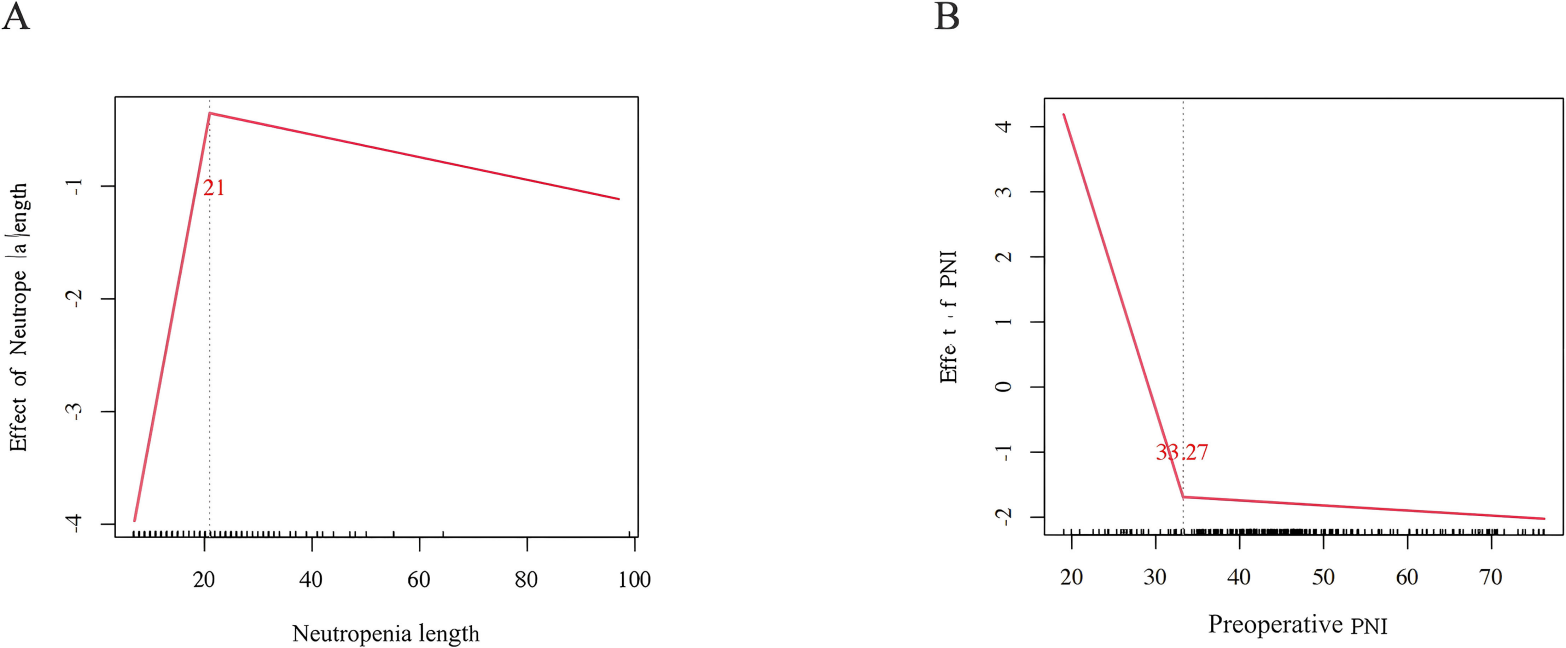
Risk threshold-guided preventive intervention strategy for perianal infection in HSCT.

**Figure 7:**
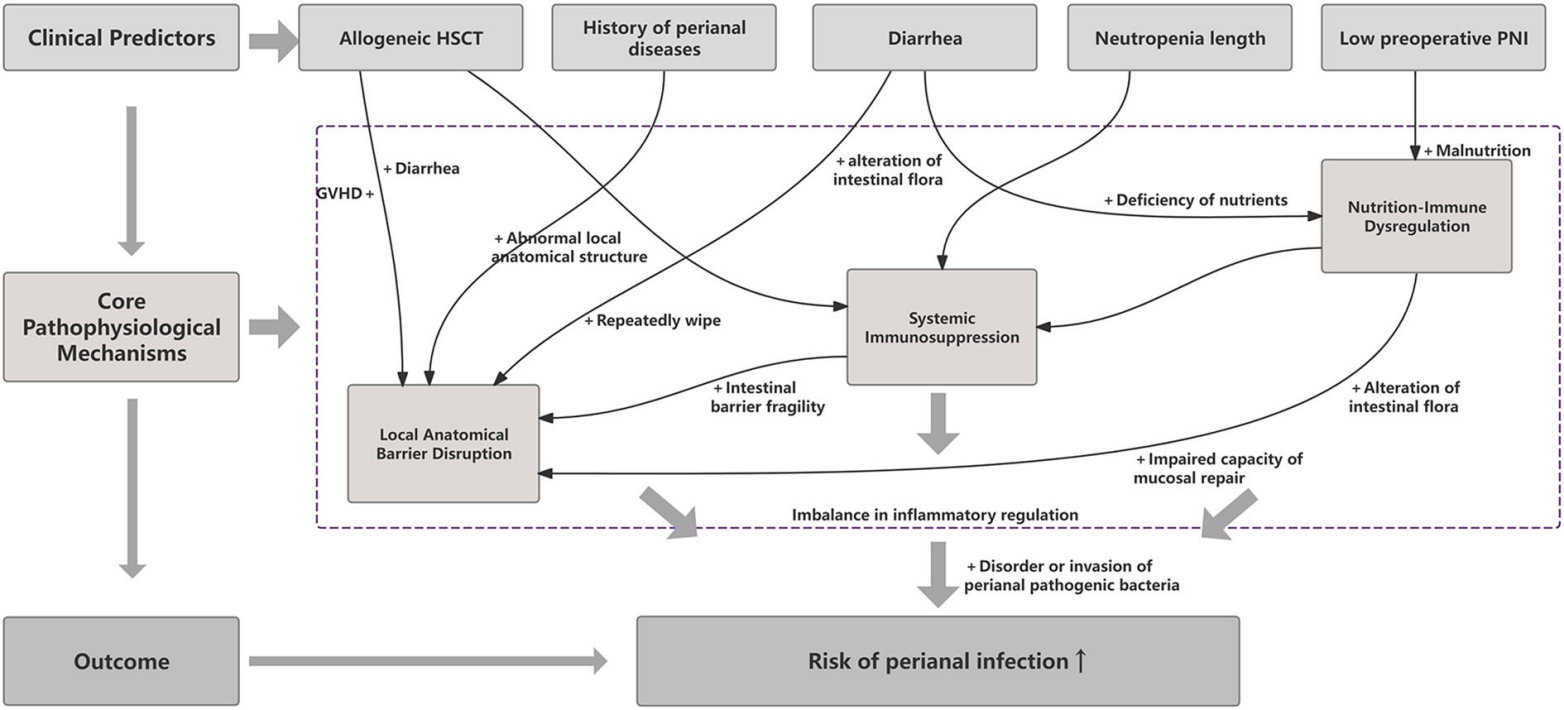
Causal pathway framework for predicting perianal infection risk following HSCT based on patient-specific factors.

Nonlinearity in the continuous variables was assessed using restricted cubic splines (RCS) with four knots, and the results are shown in **Figures 5A**–**F**, which correspond to age (A), BMI (B), neutropenia length (C), preoperative PNI (D), preoperative albumin concentration (E), and preoperative lymphocyte counts (F), respectively. Age, preoperative albumin, and preoperative lymphocyte count exhibited linear associations with perianal infection risk in the multivariable model (*p* for nonlinear >0.05). However, RCS modeling revealed significant nonlinear relationships for two key predictors. The risk of perianal infection increased nonlinearly with the neutropenia length (*p* for nonlinear<0.05; **Figure 5C**), with a marked rise in risk observed beyond a duration of 21 days (**Figure 6A**). Conversely, a nonlinear inverse association was found for the preoperative PNI (*p* for nonlinear<0.05; **Figure 5D**), where the risk demonstrated a decreasing trend when PNI exceeded 33.27 (**Figure 6B**). In these RCS models (**Figures 7A, B**), odds ratios (ORs) were adjusted for age, sex, and BMI, with knots placed at the 5th, 35th, 65th, and 95th percentiles and the 5th percentile set as the reference. Solid lines represent the ORs, and shaded areas indicate the 95% confidence intervals.

### 3.7 Causal pathway framework for predicting perianal infection risk following HSCT

In order to clarify the fundamental driving relationship between these predictive factors and the outcomes, we developed a causal pathway framework (**Figure 7**). This work provides a mechanism hypothesis to explain the potential mechanism of perianal infection in HSCT patients, demonstrating that these predictive factors may ultimately cause perianal infection in HSCT patients through a synergistic effect, under the influence of multiple intermediary factors. This integrative model situates the five key clinical predictors identified in this study within a unified pathophysiological pathway. As illustrated, these predictors (top) are proposed to operate through three interconnected core mechanisms (middle): systemic immunosuppression, local anatomical barrier disruption, and nutrition-immune dysregulation. These processes interact and converge, collectively elevating the risk of perianal infection in HSCT patients (bottom).

## 4 Discussion

This study analyzed a cohort of 353 HSCT patients, among whom 64 developed perianal infections, yielding a prevalence rate of 18.13%, which was consistent with findings from previous studies (16). Our research identified five independent predictors of perianal infection following HSCT, including the type of HSCT, history of perianal disease, diarrhea, neutropenia length, and preoperative PNI. This model, tailored specifically to the HSCT population, demonstrates acceptable performance in both discrimination and calibration. Decision curve analysis showed the model provided a net benefit at thresholds of 1-97% (training group) and 4-96% (validation group). We propose a 10% predicted risk as a pragmatic clinical cut-off. Patients below this threshold receive standard care, while those at or above it are offered an intensified prevention bundle (e.g., structured nursing assessments, sitz baths) to preempt higher-morbidity infections (5, 17). Therefore, the present model provides clinically actionable risk stratification for perianal infection following HSCT, with validated accuracy in high-risk patient identification and supports personalized clinical decision-making based on predicted outcomes.

Our study identified the type of HSCT as an independent risk factor for perianal infection following HSCT. Specifically, patients undergoing allo-HSCT exhibited a higher incidence of perianal infection compared to patients who received auto-HSCT. This increased risk is attributed to complications in allo-HSCT patients, such as delayed immune reconstitution and graft-versus-host disease (GVHD), which can increase the risk of bacterial infections (18). Furthermore, the administration of large doses of immunosuppressants in allo-HSCT compromises the skin and mucosal integrity (17). Previous studies have documented similar mucosal damage, including gastrointestinal damage, resulting from allo-HSCT pretreatment protocols (19). Besides, some chemotherapy drugs increase the risk of mucosal damage, such as methotrexate (20). Moreover, vincristine use often results in pseudo-obstruction and neurotoxicity (21). Diarrhea and hematochezia caused by acute gastrointestinal GVHD following allo-HSCT also increase the risk of perianal infection (5, 9). Moreover, research findings indicate a significantly higher death rate due to infectious complications in allo-HSCT patients compared to auto-HSCT cases (22). Therefore, the type of HSCT should be considered in the evaluation of perianal infection risk prior to HSCT, and allo-HSCT patients should be closely monitored to prevent perianal infections.

Our study identified history of perianal disease as a significant risk factor for perianal infection following HSCT. Pre-existing anorectal conditions, such as hemorrhoids or fissures, and other perianal comorbidities have been confirmed to elevate the risk of perianal infection, consistent with previous studies (5). Specifically, patients with a history of hemorrhoids were over five times more likely to develop perianal infections during chemotherapy (9), which is attributed to the presence of neutropenia and opportunistic infections after HSCT, which facilitate the progression of anorectal infections (23). Additionally, a large amount of fluid infusion and anemia-related fatigue due to chemotherapy lead to prolonged bed rest, altering defecation habits and exacerbating pre-existing hemorrhoids, potentially causing disruption and bleeding (9). Furthermore, mucosal damage also provides entry points for pathogens, thereby increasing the risk of infection. Acute GVHD following allo-HSCT often causes diarrhea and bloody stools (24), which create favorable conditions for bacterial invasion. However, the global prevalence of hemorrhoids exhibits significant regional variations, influenced by socioeconomic conditions and lifestyle factors, including dietary habits, hygiene practices, interpersonal relationships, and sexual behavior (25). Epidemiological studies indicate higher incidence rates in Asia, America, and Australia (26). For instance, in China, the prevalence is 43.7% among women and 17.7% among men (27). Similarly, in the United States, approximately one in three individuals is diagnosed with hemorrhoids during screening colonoscopy (28). Australia reports a prevalence of 38.9% (29), followed by South Korea (16.6%) (30). Consequently, pre-HSCT evaluation should include comprehensive assessment and documentation of perianal conditions, with particular attention to patients presenting with hemorrhoids or prior perianal infections. In regions with elevated hemorrhoid prevalence, we recommend implementing systematic screening protocols and prophylactic measures prior to transplantation to mitigate infection risks.

Meanwhile, our study identified diarrhea as an independent risk factor for perianal infection following HSCT, supporting previous results (5, 9). Studies revealed that up to 74.2% of individuals with hematological malignancies suffer from diarrhea while undergoing chemotherapy. Additionally, the gastrointestinal damage caused by GVHD exacerbates endotoxin migration, leading to increased inflammation and gastrointestinal damage, which primarily manifests as diarrhea and abdominal pain (31). In cases with persistent diarrhea, stool samples with deranged pH, high pancreatic lipase, bile salts, and ammonia bases can irritate the anal mucosa, causing inflammation and damage (32). Prolonged diarrhea results in the formation of phlebitis and peripheral phlebitis within the extensively stimulated perianal venous plexus, thereby weakening the vascular wall and leading to further dilation and congestion, increasing the risk of infections (9). Reducing the incidence of diarrhea during HSCT is essential. Protecting the skin surrounding the anus is important in cases with severe diarrhea.

Our predictive model revealed that the neutropenia length is related to the risk of perianal infection following HSCT. Neutrophils play a vital role in controlling infections, preventing abscess formation, and controlling inflammation (6). Specifically, the lower the neutrophil count and the longer the duration, the higher the risk of developing severe infections such as sepsis (10). However, patients with haematological malignancies often exhibit hematopoietic and immune dysfunction after chemotherapy (33). Additionally, the presence of neutropenia and opportunistic infections promotes the development of anorectal infections (23). Consequently, as the neutropenia length is extended, the incidence of infectious complications increases, particularly perianal infections (33), which is attributed to severe neutropenia, numerous chemotherapy sessions, and the utilization of broad-spectrum antibiotics, leading to the proliferation of harmful bacteria and the occurrence of gastrointestinal disorders (34). Moreover, with prolonged neutropenia, the intestinal microbiota, which plays a critical role in maintaining intestinal immune homeostasis and mucosal integrity, is affected, facilitating anorectal infection (35). In addition, studies have reported that extended neutropenia increases the risk of perianal sepsis in leukemia patients (36). Therefore, medical staff should pay particular attention to perianal skin protection during the neutropenia period after HSCT, especially when prolonged.

In this study, preoperative PNI was identified as an independent risk factor for perianal infection. The PNI reflects both nutritional and immunological status, as it is affected by serum albumin levels and lymphocyte counts (15, 37). Studies have shown that preoperative PNI can be used as a predictor of postoperative infection, complications, and prognosis, as well as tumor survival. Previous evidence suggests that a low PNI may reflect a state of malnutrition and immune dysfunction, which is detrimental to mucosal repair and increases the risk of infection. Patients with lower PNI values are exposed to a higher risk of postoperative infection and mucosal damage (14). For instance, preoperative PNI levels are inversely associated with the incidence of postoperative infectious complications in patients with esophageal tumors (e.g., anastomotic fistula, pulmonary infection) (15). Moreover, metabolites in nutrition include amino acids, fatty acids, and glucose metabolites, which are both nutrients and regulators of innate and adaptive immunity. They are regulated through signaling pathways such as free fatty acid receptors and AMP-activated protein kinase/mammalian target of rapamycin (AMPK/mTOR) (38). Notably, preoperative immunotherapy combined with chemotherapy in patients with HSCT often leads to complications such as lymphocytopenia, granulocytopenia, and hypoproteinemia. Consequently, a low preoperative PNI indicates reduced immune competence and malnutrition, often accompanied by impaired immune regulation, thereby increasing the risk of infection. Therefore, the preoperative PNI merits consideration as a significant risk indicator for perianal infection in HSCT patients.

The nonlinear relationships identified for neutropenia length and preoperative PNI add a critical layer of nuance to our risk model. The linearity assumption, while statistically convenient, may oversimplify the complex pathophysiology of post-HSCT complications. The RCS and threshold analysis confirms that the risk of perianal infection does not increase uniformly with neutropenia length but rises markedly beyond a critical threshold (approximately 21 days). This reflects the cumulative breakdown of primary mucosal defense and the escalating probability of bacterial translocation after a sustained period of profound neutropenia (35). Conversely, the nonlinear, threshold-like protective effect of PNI (with a benefit emerging above 33.27) aligns with the concept of nutritional reserves (15). It suggests that an adequate preoperative nutritional-immune status may provide a resilient buffer, potentially enhancing mucosal integrity and immune reconstitution capacity until a critical deficit is reached (14). These nonlinear patterns argue for a more nuanced, risk-stratified approach to surveillance and prophylaxis. For instance, patients anticipated to have prolonged neutropenia (>21 days) or those with a PNI below 33.27 might be prioritized for intensified perianal monitoring or pre-emptive interventions. While our study was not designed to establish definitive clinical cut-offs, these findings highlight the value of modeling nonlinear relationships to better capture biological reality and inform personalized management strategy for the perianal area in HSCT recipients.

Building upon these established factors, our study is further guided by an integrative pathophysiological framework. We propose that perianal infection risk is best understood as a convergence of three core, interconnected disturbances: systemic immunosuppression (e.g., from allogeneic HSCT and prolonged neutropenia), local epithelial barrier disruption (e.g., from prior perianal disease, diarrhea, or mucosal injury), and underlying host nutritional-immune dysregulation (reflected by indices such as the PNI). This “immune-barrier-host homeostasis” model, supported by contemporary understanding of mucosal immunology and host-microbe interactions (39). The integrative framework in our study highlights the potential mechanism of perianal infection in HSCT patients from the perspective of causal hypotheses. Allo-HSCT serves as a primary driver of profound systemic immunosuppression which through delayed immune reconstitution and GVHD, which not only directly increases infection susceptibility but can also precipitate barrier disruption (18), notably via GVHD-related diarrhea (31). A history of perianal diseases accelerates the destruction of the anatomical barrier, creating a vulnerable site for pathogen entry (5). Diarrhea further disrupts this barrier through physical maceration and altered mucosal pH, and may itself arise from other mechanisms such as GVHD (31). The neutropenia length represents a direct manifestation of impaired systemic immunity, critically weakening the primary defense against bacterial invasion (6, 10). In addition, a low preoperative PNI reflects underlying nutrition-immune dysregulation, which compromises both systemic immunocompetence and local tissue repair (14, 38), thereby exacerbating the other two mechanistic pathways. Based on these factors, the causal pathway framework we developed effectively integrates the causal relationships and synergistic mechanisms among the susceptibility factors of perianal infections after HSCT, providing a theoretical reference for the clinical implementation of preventive strategies. Therefore, moving beyond isolated risk factors, we aimed to construct a multivariate model that quantifies this integrated risk profile to provide a mechanistically grounded tool for early identification and intervention.

In summary, our study systematically evaluated and synthesized risk factors for perianal infection following HSCT and developed a visualized risk histogram to quantify individual risk. Current literature primarily focuses on influencing factors and treatment of perianal infection after HSCT, with few established risk prediction models. Yingli Wang et al. developed a prediction model for perianal infection during chemotherapy for hematological malignancies. However, this model lacks specificity to HSCT patients and may not fully capture the unique risk profile for perianal infection in this population. To address this gap, the present study constructed and validated a dedicated prediction model for perianal infection following HSCT, comprehensively quantifying associated risk factors. This prediction model demonstrated relatively robust discriminative ability, indicating clinically meaningful predictive performance, enabling clinicians and nurses to identify high-risk patients through early warning systems, implementing individualized management protocols and adaptive interventions tailored to clinical practice.

In summary, this study systematically identified and integrated key risk factors for perianal infection following HSCT into a dedicated, visualized prediction model. Existing literature has largely focused on descriptive factors or therapeutic approaches, with few models designed specifically for the HSCT population. While prior work, such as the model by Yingli Wang et al (9), for patients undergoing chemotherapy, offers valuable insights, it may not fully address the distinct pathophysiology and risk profile inherent to HSCT. Our model incorporates a causal framework and accounts for key nonlinear relationships, providing a targeted predictive tool specific to the HSCT population. It demonstrated relatively robust discriminative performance, suggesting potential utility for clinical risk stratification. This instrument could aid clinicians and nurses in early identification of high-risk patients, facilitating timely, individualized monitoring and prophylactic care within routine practice.

Nevertheless, several limitations of this study should be acknowledged. First, the single-center retrospective study with a relatively small sample size, which may introduce biases and limited its statistical power and generalizability (40, 41). Second, perianal infection was diagnosed clinically rather than by standardized imaging or microbiology, potentially introducing detection bias. Third, our cohort was restricted to patients aged 14–65 years who received myeloablative conditioning, which may limit applicability to older adults or those receiving reduced-intensity regimens populations increasingly common in contemporary HSCT practice. Consequently, the high model performance observed may partly reflect this selected population and warrants external validation in broader, more heterogeneous cohorts (42). Finally, while we propose a risk-stratified clinical decision framework based on decision curve analysis, its net benefit thresholds and corresponding interventions require prospective validation (43). Future studies should aim to include larger, prospective multicenter cohorts with standardized outcome ascertainment and systematically collect a broader range of potential predictors. Beyond expanding clinical variables, integrating biological data, such as transcriptional profiling of mucosal barriers or weighted gene co-expression network analysis (44, 45), which could uncover mechanistic links between immune-nutritional status and infection susceptibility. Combining such biomarkers with explainable artificial intelligence frameworks may further bridge clinical prediction and underlying biology, enabling more personalized risk assessment and intervention strategies.

## 5 Conclusion

In conclusion, this study identified five key predictive factors for perianal infection after HSCT, including the type of HSCT, history of perianal disease, diarrhea, neutropenia length, and preoperative PNI. Based on these findings, a visualized prediction model (histogram) incorporating a standardized scoring system was developed to enable rapid, accurate risk assessment in clinical practice. This study advances the understanding of perianal infection pathogenesis by proposing a biologically grounded mechanistic framework. Furthermore, by incorporating nonlinear effects and threshold analyses of neutropenia duration and preoperative PNI, the model provides a clinically actionable risk-stratified management strategy. This tool has certain clinical value for the early identification of patients at high risk of perianal infection after hematopoietic stem cell transplantation and for optimizing prevention strategies.

## Data Availability

The original contributions presented in the study are included in the article/supplementary material. Further inquiries can be directed to the corresponding authors.
